# Prostacyclin Analogue Beraprost Inhibits Cardiac Fibroblast Proliferation Depending on Prostacyclin Receptor Activation through a TGF β-Smad Signal Pathway

**DOI:** 10.1371/journal.pone.0098483

**Published:** 2014-05-22

**Authors:** Yun Chen, Shengju Yang, Wenjuan Yao, Hongyan Zhu, Xiaole Xu, Guoliang Meng, Wei Zhang

**Affiliations:** 1 Department of Pharmacology, School of Pharmacy, Nantong University, Nantong, Jiangsu, China; 2 Affiliated Hospital of Nantong University, Nantong, Jiangsu, China; University of Rochester Medical Center, United States of America

## Abstract

Previous studies showed that prostacyclin inhibited fibrosis. However, both receptors of prostacyclin, prostacyclin receptor (IP) and peroxisome proliferator-activated receptor (PPAR), are abundant in cardiac fibroblasts. Here we investigated which receptor was vital in the anti-fibrosis effect of prostacyclin. In addition, the possible mechanism involved in protective effects of prostacyclin against cardiac fibrosis was also studied. We found that beraprost, a prostacyclin analogue, inhibited angiotensin II (Ang II)-induced neonatal rat cardiac fibroblast proliferation in a concentration-dependent and time-dependent manner. Beraprost also suppressed Ang II-induced collagen I mRNA expression and protein synthesis in cardiac fibroblasts. After IP expression was knocked down by siRNA, Ang II-induced proliferation and collagen I synthesis could no longer be rescued by beraprost. However, treating cells with different specific inhibitors of PPAR subtypes prior to beraprost and Ang II stimulation, all of the above attenuating effects of beraprost were still available. Moreover, beraprost significantly blocked transforming growth factor β (TGF β) expression as well as Smad2 phosphorylation and reduced Smad-DNA binding activity. Beraprost also increased phosphorylation of cAMP response element binding protein (CREB) at Ser133 in the nucleus. Co-immunoprecipitation analysis revealed that beraprost increased CREB but decreased Smad2 binding to CREB-binding protein (CBP) in nucleus. In conclusion, beraprost inhibits cardiac fibroblast proliferation by activating IP and suppressing TGF β-Smad signal pathway.

## Introduction

Cardiac fibrosis, characterized by an increased collagen concentration or altered collagen composition in myocardium, enhances myocardial stiffness and hampers systolic ejection, which is a common feature in patients with advanced cardiac failure regardless of the aetiology of cardiomyopathy [1]. Therefore it is one of the major biological determinants for fatal issues in cardiovascular diseases [2]. Methods for cardiac fibrosis inhibition are rare except for some anti-hypertensive drugs [3], inhibitors of matrix matalloproteinases [4], microRNAs intervention therapies [5], and stem cell transplantations etc [6], however their effects are not satisfactory.

Prostacyclin is an endothelium-derived eicosanoid synthesized from arachidonic acid by cyclo-xygenase. It is implicated in many biological processes and is most recognized for its potent vasodilative effects, as well as its ability to inhibit aggregation of circulating platelets [7], [8]. It is quite evident that not only does prostacyclin play a key role in the vasculature, but it also contributes to the maintenance of homeostatic functions of many organ systems [9]–[12]. Potent prostacyclin releaser defibrotide markedly reduced infarct size and attenuated myocardial ischemia/reperfusion injury [13]. Beraprost limited adult rat cardiac fibroblast growth and collagen expression [14]. Iloprost inhibited transforming growth factor β (TGF-β) induced collagen deposition in dermal and NIH3T3 cells [15]. Cicaprost was effective in preventing TGF-β induced up-regulation of collagen I and other extracellular matrix related genes in mouse cardiac fibroblasts [16]. Taken together, these findings have detailed a role for prostacyclin in cardiac fibroblasts.

Prostacyclin elicits most of its cellular effects by binding to cell surface prostacyclin receptor (IP), which is a G protein-coupled receptor, thereby activating intracellular signaling pathways [17]. The main signaling linked prostacyclin binding to IP is the stimulation of adenylate cyclase (AC) via coupling to G proteins, thereby increasing intracellular cyclic AMP (cAMP) levels [18]. The biological activities of prostacyclin were believed to be exclusively mediated by IP on the cell-surface, until the cloning of peroxisome proliferator-activated receptor (PPAR) over a decade ago [19], [20]. PPARs are a family of ligand-activated nuclear receptor transcription factors. There are currently three subtypes of cloned PPARs (α, γ, β/δ), expressed in different tissues at various levels [21]–[23]. Because both IP and PPAR are abundant in cardiac fibroblasts, we investigated which receptor was vital in the anti-fibrotic effect of prostacyclin.

TGF β plays a pivotal role in the progression of myocardial fibrosis [24]. However, no information about signaling molecules downstream of TGF β was available until Smad proteins were identified [25]. On the other hand, cAMP response element binding protein (CREB) is able to be phosphorylated once intracellular cAMP level is increased by prostacyclin [26], [27]. It is known to regulate diverse stimulus-dependent transcriptional events involving collagen expression [28], [29]. Phosphorylation of CREB results in the recruitment of CREB-binding protein (CBP) in the nucleus [30], and CBP is a transcriptional co-activator and is also able to bind with Smad proteins [31]. But Chan et al found that cicaprost inhibited TGF β-induced myocardial fibrosis independent of Smad proteins [16]. Thus, the molecular mechanism involved in protective effects of prostacyclin against cardiac fibrosis remains unclear.

In the present work, we sought to investigate whether beraprost, a stable and orally active prostacyclin analogue, was potentially useful in inhibiting cardiac fibroblast proliferation. Which receptor and how the TGF β-Smad signal pathway involved in attenuation effect of beraprost were also studied.

## Materials and Methods

### Culture of cardiac fibroblasts and treatment

The investigation conformed to the Guide for the Care and Use of Laboratory Animals published by NIH and was also approved by the Institutional Animal Care and Use Committee of Nantong University. Sprague-Dawley rats, 1–3 days old, were anaesthetized with ether prior to euthanasia. Hearts were removed immediately. The ventricles were separated from the atria, trisected and digested with 0.25% trypsin (Beyotime, Haimen, China) at 37°C for 7–10 cycles until completely digested. All supernatants from each cycle, except the primal one, were pooled. Dulbecco's modified Eagle's medium (DMEM, Gibco, Carlsbad, CA) with 10% fetal bovine serum (FBS, Hyclone labs, Logan, UT) equal to supernatants were added to terminate digestion and centrifuged. The cell pellet was re-suspended in DMEM containing 10% FBS, 100 U/mL penicillin and 100 µg/mL streptomycin. Dispersed cells were incubated for 1.5 h in a 5% CO_2_ incubator. Nonmyocytes attached to the bottom of the dishes were subsequently incubated with DMEM supplemented with 10% FBS for an additional 2–4 days. Confluent cardiac fibroblasts (CFs) were treated with trypsin and subcultured. Subconfluent (>70% confluency) CFs grown in culture dishes from the second to third passage were used in the experiments. The medium was changed to DMEM supplemented with 0.5% FBS for 24 h. Confluent cells were pre-incubated with or without GW9662 (a specific antagonist of PPARγ), GSK0660 (a specific antagonist of PPARβ/δ) or GW6471 (a specific antagonist of PPARα, Sigma-Aldrich, St. Louis, MO) for 4 h. Then the cells were treated with different concentrations of beraprost (Cayman Chemical, Ann Arbor, MI, 0 µM, 2 µM, 5 µM, 10 µM, 20 µM or 40 µM) for different times (0 h, 1 h, 2 h, 4 h, 12 h or 24 h) before stimulation with Ang II (Sigma-Aldrich, St. Louis, MO, 100 nM) for an additional 24 h. Some other cells were treatment with cicaprost (Cayman Chemical, Ann Arbor, MI, 10 µM) and TGF β (PeproTech, London, UK, 5 ng/mL) simultaneously. Culture medium was used as a vehicle control.

### Cell Count Assay

Numbers of CFs was determined by cell counting Kit-8 (CCK-8, Beyotime, Haimen, China) according to the manufacturer's directions.

### Measurement of hydroxyproline

After treatment as the procedure above-mentioned, cell culture medium was collected for measuring hydroxyproline content according to our previous research [3].

### RNA interference

Three double-strand RNA oligos for specific IP siRNA (siRNA #1 sense, 5′-CACGAGAGGAUGAAGUUUATT-3′, antisense, 5′-UAAACUUCAUCCUCUCGUGTT-3′; siRNA#2 sense, 5′- GCCUUCGCUAUGACUUUCUTT-3′, antisense, 5′-AGAAAGUCAUAGCGAAGGCTT-3′; siRNA #3 sense, 5′-CCGAUUCUCUCCAGGCUAATT-3′, antisense, 5′-UUAGCCUGGAGAGAAUCGGTT-3′) were synthesized (GenePharma, Shanghai, China). Commercially available siRNA to random noncoding sequences were used for nonspecific control siRNA (NC siRNA).

The cultured CFs were serum deprived for 4 h and then transfected with IP siRNA or NC siRNA using the Lipofectamine 2000 reagent (Invitrogen, Carlsbad, CA) according to the manufacturer's protocol. Twenty-four hours after transfection, cells were pre-treated with beraprost (10 µM) for 4 h followed by Ang II (100 nM) for 24 h.

### Quantitative real time polymerase chain reaction

Quantitative real time PCR analysis was used to measure mRNA expression with 18S set as a control. Total RNA was extracted using Trizol reagent (Takara, Otsu, Shiga, Japan). RNA (500 ng) was added as a template to reverse-transcriptase reactions carried out using the PrimeScript RT Master Mix Kit (Takara, Otsu, Shiga, Japan). PCRs were carried out with the resulting cDNAs using the SYBR Green Premix (Takara, Otsu, Shiga, Japan) with ABI 7500 Real Time PCR System (ABI, Carlsbad, CA). Experimental Ct values were normalized to 18S and relative mRNA expression was calculated versus a reference sample. Each sample was run and analyzed in triplicate. Primers used to amplify the fragment of collagen I, collagen III, TGF β, α-smooth muscle actin (α-SMA) and 18S were [32]–[34]: collagen I: 5′-AGGGTCATCGTGGCTTCTCT-3′ and 5′-CAGGCTCTTGAGGGTAGTGT-3′; collagen III: 5′-AGCGGAGAATACTGGGTTGA-3′ and 5′-GATGTAATGTTCTGGGAGGC-3′; TGF β: 5′-GCCCTGGACACCAACTATTGC-3′ and 5′-GGAGCGCACGATCATGTTGG-3′; α-SMA: 5′-CATCAGGAACCTCGAGAAGC-3′ and 5′-TCGGATACTTCAGGGTCAGG-3′; 18S, 5′-AGTCCCTGCCCTTTGTACACA -3′ and 5′- GATCCGAGGGCCTCACTAAAC -3′.

### Western blot analysis

Protein samples were separated on sodium dodecyl sulfate polyacrylamide gel electrophoresis (SDS-PAGE), transferred onto polyvinylidene fluoride (PVDF) membrane (Millipore, Billerica, MA). After blocking at room temperature in 5% w/v non-fat milk with TBST buffer (Tris-HCl 10 mM, NaCl 120 mM, Tween-20 0.1%; pH 7.4) for 2 h, membranes were incubated overnight with the appropriate primary anti-collagen I, anti-collagen III, anti-TGF β, anti-Smad2, anti-p-Smad2, anti-CBP, anti-Ser142-p-CREB, anti-Ser133-p-CREB (1∶500, Bioworld Technology, St. Louis Park, MN), anti-tubulin, anti-Lamin B1(1∶1000, Santa Cruz Biotechnology, Santa Cruz, CA), anti-GAPDH (1∶6000, Sigma-Aldrich, St. Louis, MO) at 4°C and then incubated with horseradish peroxidase (HRP)-conjugated secondary antibody at room temperature for 2 h. Proteins were visualized by enhanced chemiluminescence substrate (ECL, Pierce, Rockford, IL).

### Measurement of Smad binding activity

To detect Smad DNA binding activity, a 5′-biotin-labeled oligonucleotide probe containing 3 of the Smad-binding CAGA box motif were synthesized. Smad binding activity was measured using an electrophoretic mobility shift assays (EMSA) kit (Beyotime, Haimen, China) following the manufacturer's instructions. Nucleotide sequences of the oligonucleotides used for EMSA were 5′- GAGGTAGCCAGACAGGTAGCCAGACAGGGAGCCAGACAG -3′ with biotin-labeled at the 5′ end (Invitrogen, Carlsbad, CA). In brief, nuclear proteins extracted from cardiac fibroblasts were prepared using a Nuclear and Cytoplasmic Extraction Reagents Kit (Thermo Fisher Scientific Inc., Rockford, IL). Nuclear protein (8 µg) samples were incubated for 20 min at room temperature with 5×binding buffer (2 µL) and Smad oligonucleotide probe (1 µL). Probe without biotin-labeling served as a nonspecific competitor was added to the mixture prior to labeled probe addition for 20 min. DNA-protein complexes were resolved on non-denaturing acrylamide gels (4%) and transferred to a nylon membrane (Amersham Pharmacia Biotech, Buckinghamshire, UK). The proteins were twice ultraviolet cross-linked for 1 min each time. Protein bands were visualized by ECL procedure.

### Co-immunoprecipitation

Equal amount of nuclear protein lysate with protease inhibitor cocktail (Roche, Indianapolis, IN) from CFs was tumbled with Protein G beads (Roche, Indianapolis, IN) and pre-cleared for 30 min at room temperature. After centrifugation (20 s, 12,000 g), the pre-cleared lysate was allowed to tumble with anti-CBP antibody for 1 h prior to the addition of Protein G beads. The conjugated beads and lysate were tumbled overnight at 4°C. The beads were washed with two separate washing buffers (Buffer A: 50 mM Tris-HCl pH 7.5, 150 mM NaCl, 1% Tween-20, 0.05% Na Deoxycholate; Buffer B: 50 mM Tris-HCl pH 7.5, 75 mM NaCl, 0.1% Tween-20) for 4 times. Protein was eluted from the beads using 20 µL of 2×4% SDS sample buffer with β-mercaptoethanol (2.5% final concentration) and heated to 100°C prior to for further western blot analysis.

### Immunofluorescence staining

CFs were pre-treated with beraprost (10 µM) for 4 h before being incubated with or without Ang II (100 nM) for 24 h. Then the cells were blocked with 10% bovine serum albumin (Solarbio, Beijing, China) and incubated with primary antibody against α-SMA (1∶1000, Santa Cruz Biotechnology, CA), collagen I or negative IgG control for 16 h at 4°C. Immunoreactivity was visualized using Alexa Fluor 488 conjugated IgG (Beyotime, Haimen, China, 1∶1000). Cells were counterstained with DAPI (5 µg/mL, Beyotime, Haimen, China) and then evaluated under a fluorescence microscope (Nikon, Tokyo, Japan).

### Statistical Analysis

The data are expressed as mean ± standard error of mean (SEM) and analyzed using one way ANOVA with Tukey's post-test analysis for comparison of intra as well as inter-group variance. Statistical significance was assumed when *P*<0.05.

## Results

### Beraprost inhibited Ang II-induced cardiac fibroblast proliferation

**Figure 1:**
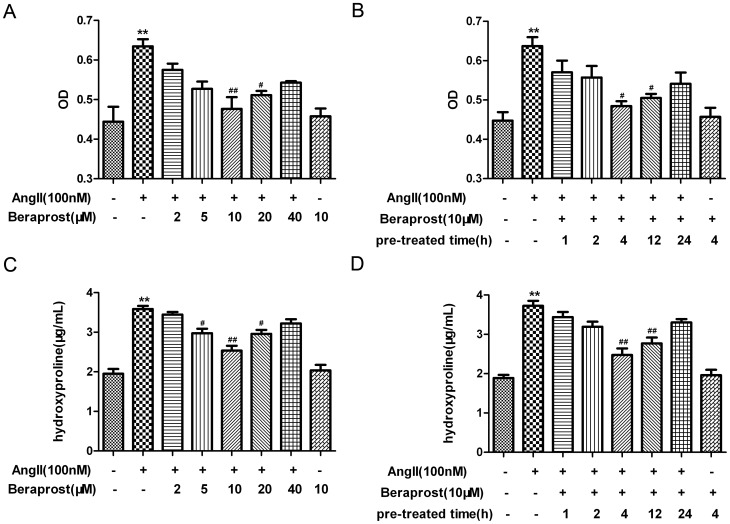
Beraprost inhibited Ang II-induced cardiac fibroblasts proliferation. (A) Neonatal rat cardiac fibroblasts were pre-treated with different concentrations of beraprost for 4 h followed by Ang II (100 nM) stimulation for an additional 24 h. The number of cells was represented as an OD value using a cell count assay. (B) Content of hydroxyproline in cell culture medium was determined. (C) Neonatal rat cardiac fibroblasts were pre-treated with beraprost (10 µM) for different times followed by Ang II (100 nM) stimulation for an additional 24 h. The number of cells was represented as an OD value. (D) Content of hydroxyproline in cell culture medium was determined. Values are expressed as mean ± SEM. Cells treated with culture medium served as a vehicle control (con). ^**^ *P*<0.01, compared with con, ^#^ *P*<0.05, ^##^ *P*<0.01 compared with only Ang II stimulated group. n = 4–5.

To determine whether prostacyclin inhibits CFs proliferation, neonatal rat CFs were pre-incubated with different concentrations of beraprost for 4 h and then exposed to Ang II (100 nM) for an additional 24 h. The number of cardiac fibroblasts was evaluated by cell count analysis (represented as an OD value) and content of hydroxyproline. Ang II significantly increased the number of CFs and hydroxyproline concentration in the medium. Pre-incubation with beraprost (5 µM, 10 µM or 20 µM) for 4 h significantly inhibited proliferation of cardiac fibroblasts, among which the effect was maximized at 10 µM of beraprost and therefore this concentration was chosen for following *in vitro* treatments. It was observed that beraprost effectively protected against Ang II-induced proliferation of CFs in a concentration-dependent manner. The time course for beraprost (10 µM) pre-treatment was then optimized. Similar experiments indicated that pre-incubation with beraprost (10 µM) for 4 h or 12 h remarkably inhibited proliferation of CFs induced by Ang II (Fig. 1C and 1D). This suggested that beraprost was effective in suppressing Ang II-induced cardiac fibroblast proliferation in a time-dependent manner. Moreover, the attenuating effect peaked at 4 h of beraprost pre-exposure, which was chosen for further extensive study. No significant alteration of cell numbers or hydroxyproline levels was detected after beraprost pre-treatment without Ang II stimulation (Fig. 1).

### Beraprost suppressed Ang II-induced collagen synthesis in cardiac fibroblasts

**Figure 2:**
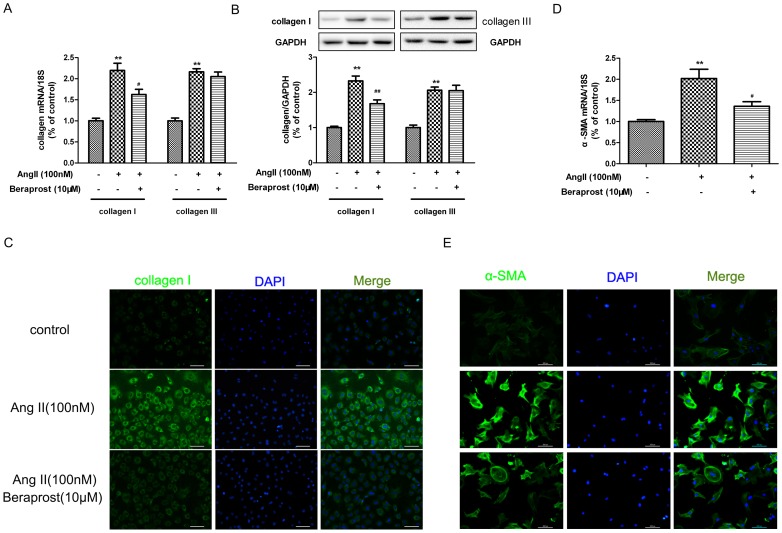
Effects of beraprost on Ang II-induced collagen synthesis in cardiac fibroblasts. Neonatal rat cardiac fibroblasts were pre-treated with beraprost (10 µM) for 4 h followed by Ang II (100 nM) stimulation for an additional 24 h. (A) Collagen I and collagen III mRNA expressions were assessed by real time PCR. (B) Cell lysates were tested for collagen I and collagen III protein expression by western blot. (C) Cellular collagen I was visualized using Alexa Fluor 488 conjugated IgG by immunofluorescence staining. The nuclei were counter-stained with DAPI (Scale bar: 100 µm). (D) Expression of α-SMA mRNA was assessed by real time PCR. (E) Cellular α-SMA was visualized using Alexa Fluor 488 conjugated IgG by immunofluorescence staining. The nuclei were counter-stained with DAPI (Scale bar: 100 µm). Values are expressed as mean ± SEM. Cells treated with culture medium served as a vehicle control (con). ^**^ *P*<0.01, compared with con, ^#^ *P*<0.05, ^##^ *P*<0.01 compared with only Ang II stimulated group. n = 4.

Enhancement of collagens, type I and type III, is the predominant phenotype in cardiac fibrosis [35]. We therefore examined whether beraprost suppressed collagen synthesis after Ang II stimulation. Compared with medium-treated vehicle control, Ang II significantly increased expression of collagen I and III at both mRNA and protein level. Pre-treatment with beraprost (10 µM, 4 h) down-regulated collagen I expression (Fig. 2A and 2B), but levels of collagen III showed no obvious alteration after beraprost administration. Therefore the effect of beraprost on collagen III was disregarded throughout the remainder of the study. Immunofluorescence staining was carried out to further validate that beraprost inhibited collagen I expression in CFs (Fig. 2C). It is somewhat puzzling that the change of collagen III expression is not consistent with that of collagen I expression after beraprost pre-treatment. Expression of α-SMA, one of the most independent and robust markers of myofibroblast differentiation [36], was detected by real time PCR and immunofluorescence. It was found that expression of α-SMA at both mRNA and protein level enhanced significantly after Ang II stimulation, which was decreased with beraprost (10 µM, 4 h) pre-treatment (Fig. 2D–E). Taken together, these accordant results confirmed that beraprost suppressed Ang II-induced collagen synthesis in CFs.

### Beraprost attenuated Ang II-induced proliferation and collagen I synthesis in cardiac fibroblasts in an IP-dependent manner

**Figure 3:**
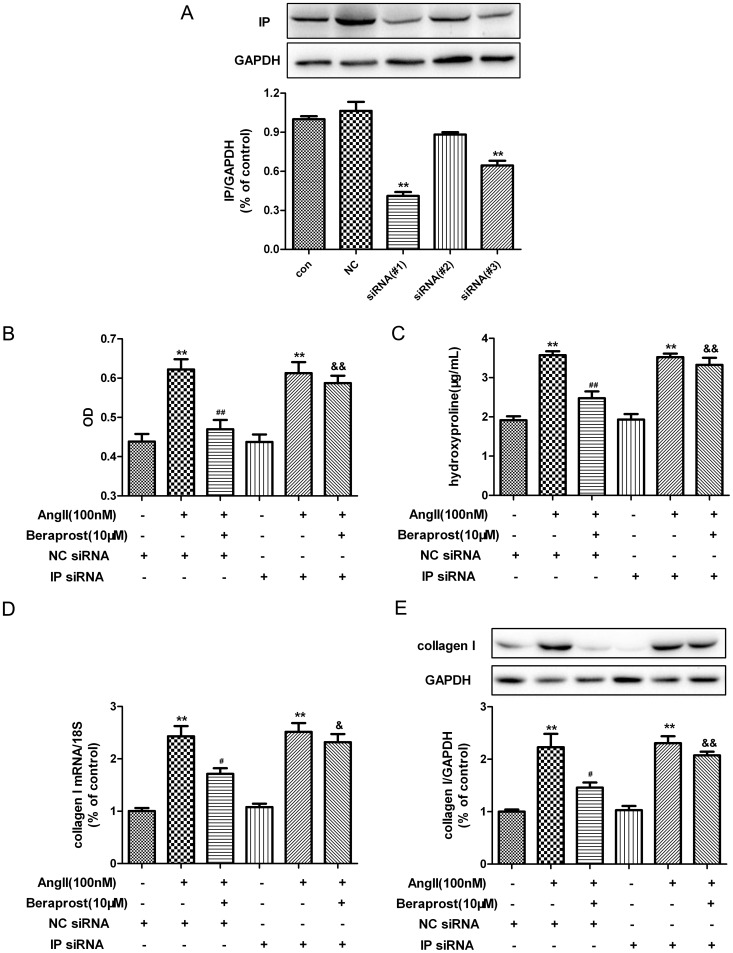
Prostacyclin receptor (IP) contributes to beraprost-mediated attenuating effect on cardiac fibroblast proliferation and collagen I synthesis. (A) Neonatal rat cardiac fibroblasts were serum deprived for 4 h and then transfected with IP-specific siRNA (siRNA#1, siRNA#2 or siRNA #3) or nonspecific control siRNA (NC siRNA) for 24 h. Cell lysates were tested for IP expression by western blot. Cells treated with culture medium or NC siRNA served as a vehicle control (con) or nonspecific control (NC). ^**^ *P*<0.01, compared with NC. (B) SiRNA transfected cells were pre-treated with beraprost (10 µM) for 4 h followed by Ang II (100 nM) stimulation for an additional 24 h. The number of cells represented as an OD value using a cell count assay. (C) Content of hydroxyproline in cell culture medium was determined. (D) Collagen I mRNA expression was assessed by real time PCR. (E) Cell lysates were tested for collagen I protein expression by western blot. Values are expressed as mean ± SEM. ^**^ *P*<0.01, compared with corresponding siRNA transfected group, ^#^ *P*<0.05, ^##^ *P*<0.01 compared with NC siRNA transfected followed by Ang II stimulated group, ^&^ *P*<0.05, ^&&^ *P*<0.01, compared with NC siRNA transfected followed by beraprost pre-treatment and Ang II stimulated group. n = 4-5.

Two receptors, IP in cell membrane and PPAR in nucleus, might be responsible for the above attenuating effects. Therefore we next assessed the contributions of IP in CFs after beraprost and Ang II stimulation by silencing IP with siRNA technology. Firstly, three kinds of siRNA sequences specific for IP were designed and transfected into CFs respectively. The efficiency of siRNA was assessed with western blot. Expression of IP significantly reduced in CFs with siRNA#1 and siRNA#3 transfection. And expression of IP decreased to approximately one third of the level of control cells after CFs were transfected with siRNA#1, which showed best effect on IP knockdown and was chosen for further experiments (Fig. 3A). Then we examined the anti-proliferative effect of beraprost on CFs after IP silencing following Ang II treatment for an additional 24 h. Our study found that Ang II increased proliferation (assessed as cell numbers and hydroxyproline content, Fig. 3B and 3C) and collagen I synthesis (determined by real time PCR and western blot, Fig. 3D and 3E), and beraprost inhibited Ang II-induced proliferation and collagen I synthesis after cardiac fibroblasts were transfected with NC siRNA. However, cell numbers, hydroxyproline content and collagen I expression in Ang II/beraprost/IP siRNA group were higher than that in Ang II/Beraprost/NC siRNA group, which was similar to the level in Ang II/NC siRNA group or Ang II/IP siRNA group. It suggested that Ang II-induced cardiac fibroblast proliferation could no longer be ‘rescued’ by beraprost after IP was knocked down. Taken together these results indicate that IP is essential for the anti-proliferative effect of beraprost on Ang II-stimulated CFs.

### PPAR was not involved in the anti-proliferative effect of beraprost on Ang II-stimulated cardiac fibroblasts

**Figure 4:**
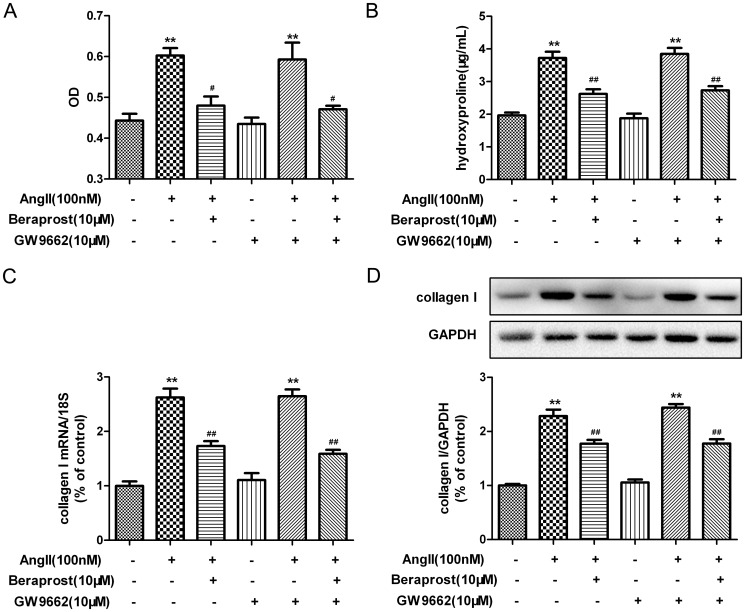
Peroxisome proliferators-activated receptor γ (PPARγ) is not involved in attenuating effect of beraprost on Ang II-induced cardiac fibroblast proliferation and collagen I synthesis. Neonatal rat cardiac fibroblasts were serum deprived for 24-treated with specific PPARγ antagonist GW9662 (10 µM) for 4 h. Cells were then incubated with beraprost (10 µM) for 4 h followed by Ang II (100 nM) stimulation for an additional 24 h. (A) The number of cells was represented as an OD value using a cell count assay. (B) Content of hydroxyproline in cell culture medium was determined. (C) Collagen I mRNA expression was assessed by real time PCR. (D) Cell lysates were tested for collagen I protein expression by western blot. Values are expressed as mean ± SEM. Cells without Ang II and beraprost stimulation served as a control (con). ^**^ *P*<0.01, compared with con, ^#^ *P*<0.05, ^##^ *P*<0.01 compared with Ang II stimulated group following with or without GW9662 pre-treatment. n = 4–6.

**Figure 5:**
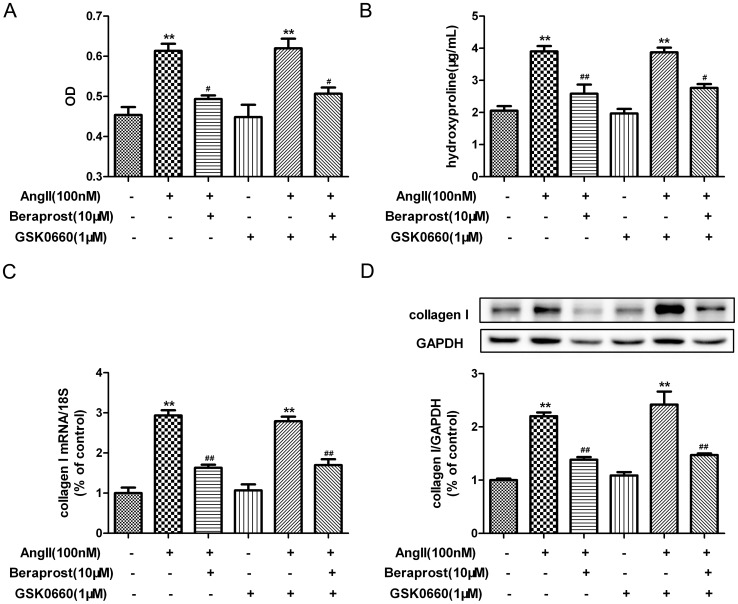
Peroxisome proliferators-activated receptor β/δ (PPARβ/δ) is not involved in attenuating effect of beraprost on Ang II-induced cardiac fibroblast proliferation and collagen I synthesis. Neonatal rat cardiac fibroblasts were serum deprived for 24-treated with specific PPARβ/δ antagonist GSK0660 (1 µM) for 4 h. Cells were then incubated with beraprost (10 µM) for 4 h followed by Ang II (100 nM) stimulation for an additional 24 h. (A) The Nnumber of cells was represented as an OD value using a cell count assay. (B) Content of hydroxyproline in cell culture medium was determined. (C) Collagen I mRNA expression was assessed by real time PCR. (D) Cell lysates were tested for collagen I protein expression by western blot. Values are expressed as mean ± SEM. Cells without Ang II and beraprost stimulation served as a control (con). ^**^ *P*<0.01, compared with con, ^#^ *P*<0.05, ^##^ *P*<0.01 compared with Ang II stimulated group following with or without GSK0660 pre-treatment. n = 3–4.

**Figure 6:**
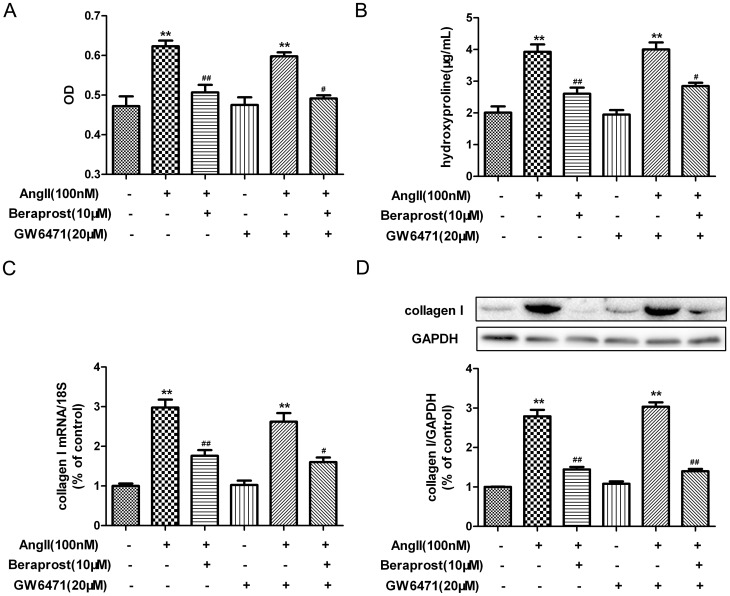
Peroxisome proliferators-activated receptor α (PPARα) is not involved in attenuating effect of beraprost on Ang II-induced cardiac fibroblast proliferation and collagen I synthesis. Neonatal rat cardiac fibroblasts were serum deprived for 24-treated with specific PPARα antagonist GW6471 (20 µM) for 4 h. Cells were then incubated with beraprost (10 µM) for 4 h followed by Ang II (100 nM) stimulation for an additional 24 h. (A) The number of cells was represented as an OD value using a cell count assay. (B) Content of hydroxyproline in cell culture medium was determined. (C) Collagen I mRNA expression was assessed by real time PCR. (D) Cell lysates were tested for collagen I protein expression by western blot. Values are expressed as mean ± SEM. Cells without Ang II and beraprost stimulation served as a control (con). ^**^ *P*<0.01, compared with con, ^#^ *P*<0.05, ^##^ *P*<0.01 compared with Ang II stimulated group following with or without GW6471 pre-treatment. n = 3–4.

PPAR was previously identified as a putative receptor responsible for the modulation of target gene expression in response to prostacyclin analogues [20]. In order to determine whether PPAR is involved in the attenuating effects of beraprost on Ang II-induced CFs proliferation and collagen I synthesis, CFs were pre-incubated with GW9662 (a specific antagonist of PPAR γ, 10 µM) [37] for 4 h following with beraprost treatment and Ang II stimulation. Similar suppressive ability of beraprost on cell number and hydroxyproline secretion was still detected after GW9662 pre-treated, while GW9662 treatment alone didn't show any biological effect on CFs proliferation (Fig. 4A and 4B). Similarly, GW9662 didn't ablate the reduction ability on collagen I content at either mRNA or protein level (Fig. 4C and 4D). Two more antagonists specific to PPARβ/δ (GSK0660, 1 µM) [38] and PPARα (GW6471, 20 µM) [39] were used in further experiments. Similar to GW9662, pre-treatment with GSK0660 or GW6471 prior to beraprost incubation and Ang II stimulation didn't ablate the reduction on cardiac fibroblast proliferation and collagen synthesis (Fig. 5 and Fig. 6). All of these findings suggested that PPAR might not be involved in the anti-proliferative effect of beraprost on Ang II-stimulated cardiac fibroblasts.

### Beraprost blocked TGF β-Smad signal pathway in Ang II-stimulated cardiac fibroblasts

**Figure 7:**
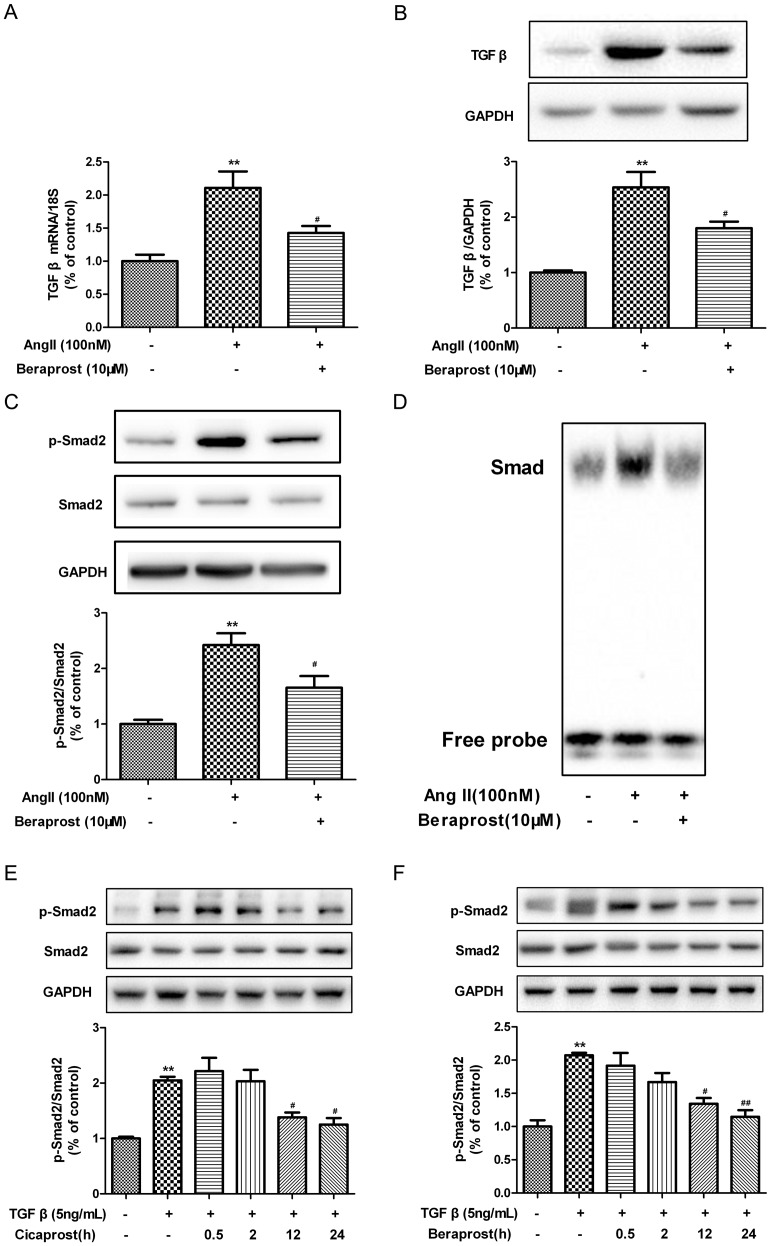
Beraprost blocked TGF β-Smad signal pathway in Ang II-induced cardiac fibroblasts. (A) Neonatal rat cardiac fibroblasts were pre-treated with beraprost (10 µM) for 4 h followed by Ang II (100 nM) stimulation for an additional 24 h. TGF β mRNA expression was assessed by real time PCR. (B) Cell lysates were tested for TGF β protein expression by western blot. (C) Phosphorylation of Smad2 was determined by western blot. (D) Smad-DNA binding activity was examined with a DNA probe containing 3 of the Smad-binding CAGA box motif using electrophoresis mobility shift assay (EMSA). (E) Neonatal rat cardiac fibroblasts were treated with TGF β (5 ng/mL) and cicaprost (10 µM) simultaneously for different time. Cell lysates were tested for Smad2 protein expression by western blot. (F) Neonatal rat cardiac fibroblasts were treated with TGF β (5 ng/mL) and beraprost (10 µM) simultaneously for different time. Cell lysates were tested for Smad2 protein expression by western blot. Values are expressed as mean ± SEM. Cells treated with culture medium served as a vehicle control (con), ^**^ *P*<0.01, compared with con, ^#^ *P*<0.05, ^##^ *P*<0.01 compared with only Ang II or TGF β stimulated group. n = 3–5.

TGF β-Smad signal pathway is activated and facilitated the progression of myocardial fibrosis [40]. Expression of TGF β and Smad2, two critical members of TGF β-Smad molecular signal pathway, was detected. As expected, exposure of CFs to Ang II (100 nM, 24 h) enhanced expression of TGF β and phosphorylation of Smad2. Moreover, treatment with beraprost significantly decreased both TGF β mRNA and protein expression (Fig. 7A and 7B). Beraprost also reduced Smad2 phosphorylation while total Smad2 levels remained stable (Fig. 7C). Data suggested that TGF β-Smad2 pathway was activated during Ang II-mediated CFs proliferation, which was likely to be suppressed by beraprost. We also assessed the DNA binding activity of fibroblast nuclear proteins using a probe containing 3 of the Smad-binding CAGA box motif. A stronger binding activity to Smad-binding sites was detected in nuclear proteins from Ang II-stimulated cells, whereas beraprost weakened this binding activity (Fig. 7D). We noted that cicaprost, another prostacyclin analogue, inhibited collagen and SM-22 but did not inhibit Smad phosphorylation over 120 min with TGF β stimulation in previous study by Chan et al [16]. It was not consistent with our finding that beraprost did inhibit Smad phosphorylation at 24 h with Ang II stimulation. In order to make clear that whether this contradiction was truly a difference in mechanism of these two compounds, or was due to different timing or other factors, we repeated the experiments with two prostacyclin analogues respectively. We found that cicaprost as well as beraprost could not inhibit Smad2 phosphorylation at 0.5 h or 2 h with TGF β stimulation, which agreed with Chan's report [16]. However, both compounds significantly decreased Smad2 phosphorylation at 12 h and 24 h (Fig. 7E–F).

### Beraprost regulated CREB phosphorylation and nuclear translocation in Ang II-stimulated cardiac fibroblasts

**Figure 8:**
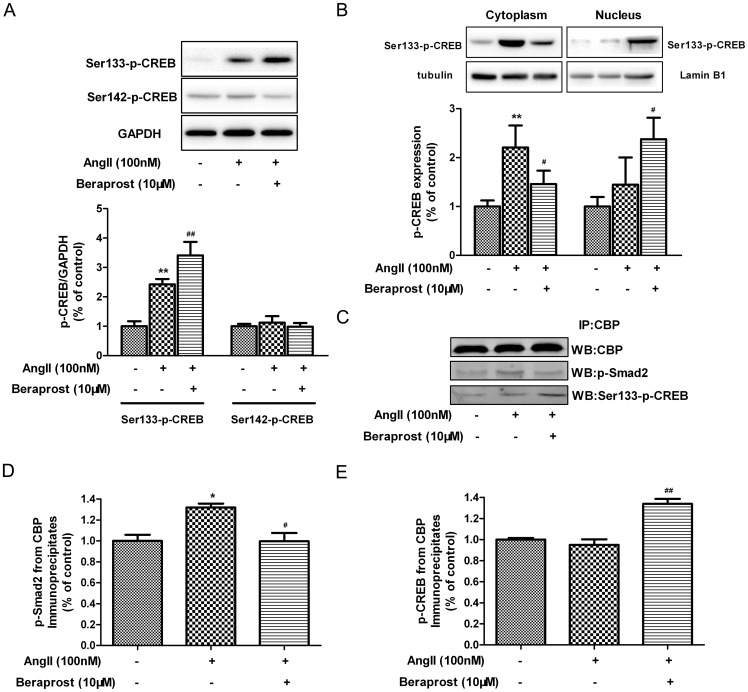
Beraprost regulated CREB phosphorylation and nuclear translocation in Ang II-induced cardiac fibroblasts. Neonatal rat cardiac fibroblasts were pre-treated with beraprost (10 µM) for 4 h followed by Ang II (100 nM) stimulation for an additional 24 h. (A) Cell lysates were tested for phosphorylation of CREB at Ser133 and Ser142 by western blot. (B) Proteins from cytoplasm and nucleus respectively were tested for phosphorylation of CREB at Ser133 by western blot. (C) Nuclear proteins were immunoprecipitated with anti-CBP antibody. The precipitated proteins were analyzed with western blots using anti-Smad2 or Ser133-p-CREB antibodies. The total CBP detected with the anti-CBP antibody was used to normalize the signals. (D) Quantitative analysis of p-Smad2-CBP association. (E) Quantitative analysis of p-CREB-CBP association. Values are expressed as mean ± SEM. Cells treated with culture medium served as a vehicle control (con), ^*^ *P*<0.05, ^**^ *P*<0.01, compared with con, ^#^ *P*<0.05, ^##^ *P*<0.01, compared with only Ang II stimulated group. n = 4.

Prostacyclin, a cAMP-elevating agent, can induce phosphorylation of CREB [16]. Next we determined the influence of beraprost on the phosphorylation of CREB followed with Ang II stimulation. Ang II significantly increased phosphorylation of CREB at Ser133 (a requirement for its activation), but not Ser142, which made phosphorylation site of Ser133 the focus of mechanism in further study. Interestingly, there was an additional enhancement of phosphorylation of CREB at Ser133 after beraprost pre-treatment (Fig. 8A). Although CREB educes potential biological activity only once it enters into nucleus to bind with CBP, whether prostacyclin regulated p-CREB nuclear translocation with the present of Ang II is vague. Thus proteins extracted from cytoplasm and nucleus respectively in CFs were quantified with western blot. We found that phosphorylation of CREB at Ser133 in cytoplasm enhanced significantly after Ang II stimulation, accompanied with its translocation into nucleus in response to beraprost (Fig. 8B). Both CREB and Smad are able to bind with CBP in the nucleus, and our co-immunoprecipitation analysis of nuclear protein indicated that more CREB but less Smad2 binding with CBP after beraprost pre-treatment (Fig. 8C–E). It suggested that increased p-CREB in the nucleus after beraprost treatment sequestrated the transcription co-activator CBP and then prevented Smad-related transcription, which might augment the inhibition of beraprost on TGF β-Smad signal pathway.

## Discussion

For many decades prostacyclin has been recognized as a key player in cardiovascular homeostasis, with numerous studies demonstrating a clear role for prostacyclin in the pathologic response of fibrosis. Beraprost, one common prostacyclin analogue, selectively inhibits proliferation in a dose-dependent manner in murine primary pulmonary arterial smooth muscle cells [41]. ONO-1301, a synthetic prostacyclin agonist, suppressed myofibroblast expansion and liver fibrosis in CCl_4_-induced mice [42]. ONO-1301 also improved airway remodeling induced by ovalbumin in mice [43], but little is known about the role of prostacyclin in myocardial fibrosis. A previous study reported that Ang II stimulated greater expression of prostacyclin in cultured cardiac fibroblasts of Wistar-Kyoto rats (WKY) rather than in spontaneously hypertensive rats (SHR) [14]. Beraprost decreased growth rate and DNA synthesis of fibroblasts and inhibited collagen expression in WKY cells, which is less responsive in SHR cells [14]. Long-term prostacyclin administration preserved diastolic function and prevented myocardial interstitial fibrosis in the hypertension model of salt-sensitive Dahl rats [44]. However, whether beraprost could attenuate Ang II-induced cardiac fibroblasts proliferation was unknown. In our study, beraprost pre-incubation significantly inhibited cardiac fibroblasts proliferation and collagen synthesis induced by Ang II in a dose-dependent and time-dependent manner, suggesting its potential effect in restraining cardiac fibrosis induced by Ang II.

Prostacyclin selectively activates the cell membrane G protein-coupled receptor, known as the prostacyclin receptor (IP), to carry out its physiological actions [17]. Deficiency in IP contributes to atherothrombosis, as well as higher severity in patients with coronary artery disease, suggesting that IP appears to play a critical role in modulating coronary heart disease [45]. A previous study found that IP played a suppressive role in the development of pressure overload-induced cardiac hypertrophy [46]. In the whole animal, several investigators reported that compared to wild type littermates, the IP^−/−^ mice developed severer cardiac fibrosis (perivascular and interstitial locales) as a result of myocardial infarction, aortic banding induced hypertension and salt sensitive hypertension [44], [46], [47]. Ang II-induced cardiac fibrosis was also exacerbated in ApoE^−/−^/IP^−/−^ double knockout mice with hyperlipidemia [16]. Until now, whether IP is indispensable for the inhibition of prostacyclin on cardiac fibrosis is vague. In our study, the attenuating effect of beraprost on proliferation and collagen synthesis was abolished when IP was potently knocked down in cardiac fibroblasts, suggesting that the protective effect of prostacyclin against cardiac fibrosis depends, at least partly, on IP.

Carcinogen-induced lung tumor incidence is similar in IP^+/+^, IP^+/−^ and IP^−/−^ mice, indicating that these protective effects of prostacyclin are not mediated through activation of IP. Further study identified PPARγ as a critical target for prostacyclin-mediated lung cancer chemoprevention [48]. Prostacyclin also induced the translocation of PPARα from the cytosol into the nucleus and attenuated NF-κB-induced TNF-α activation following renal ischemia/reperfusion (I/R) injury [49]. The protection of prostacyclin on vascular remodeling also appeared to be mediated, at least in part, by activation of PPARδ [19]. Adiponectin protected renal I/R injury in a prostacyclin-PPARα-dependent signaling pathway [50]. Iloprost, based on its chemical structural, was indicated as a dual PPARα/δ agonist [51]. Studies also verified that prostacyclin induced synthetic-to-contractile phenotypic modulation in smooth muscle cells through the activation of PPARα/δ [52]. Others found that acute prostacyclin-induced calcium-dependent potassium channels (K_Ca_) activation was critically dependent on PPARβ/δ as a rapid signaling factor in human pulmonary arterial smooth muscle cells [20]. Teunissen et al showed that activation of PPARδ suppressed the proliferation of neonatal rat cardiac fibroblasts [53], while Lin et al demonstrated that beraprost induced the activation of PPARδ in vascular smooth muscle cells [54]. These discrepancies might be explained by different types of cells, different stimulus, different drugs with discrepant profile of pharmacokinetics, different treatment durations used in such treatments, or a combination of these factors. Altogether, PPAR was identified as a putative receptor involved in the biological effects of prostacyclin analogues. All three receptors have been detected in cardiac fibroblast [55]–[57], and we found that three antagonists specific for PPARγ, PPARβ/δ or PPARα respectively showed no significant alleviation on beraprost's inhibitive effect on reduplication of cardiac fibroblast induced by Ang II stimulation. It is suggested that prostacyclin protects against cardiac fibrosis in a PPAR-independent manner. However, combination of beraprost and PPAR antagonist might be not valuable in clinical application because of the absence of synergetic effects if administrated together.

It has also been shown that prostacyclin regulated TGF β-Smad pathway in multiple cell lines or tissues. Treatment with synthetic prostacyclin agonist decreased the expressions of TGF β in liver fibrosis [42]. Prostacyclin also significantly suppressed the increase of TGF β expression and Smad2/3 phosphorylation in kidney [58]. Smooth muscle cell proliferation could also be inhibited by prostacyclin with enhanced Smad1/5 phosphorylation [59]. Meanwhile beraprost inhibited TGF β-induced Smad-dependent and Smad-independent signaling via protein kinase A-dependent pathway by reducing the phosphorylation of Smad2, Smad3 and p38 mitogen-activated protein kinase proteins [41]. However, it has also been reported that prostacyclin derivatives prevented the fibrotic response to TGF β without any influence on Smad signaling in fibroblasts [15], and TGF β-mediated activation of the Smad pathway in cardiac fibroblasts was unlikely to be directly modulated by cicaprost [16]. Above-mentioned contradictory findings on TGF β-Smad pathways after prostacyclin treatment might be attributed to the differences in fibrotic models, distinguish characteristic and duration of prostacyclin agonist. More importantly, we found that beraprost induced a significant decrease in TGF β expression and Smad2 phosphorylation in Ang II-stimulated cardiac fibroblasts. Alleviated binding activity of Smad to DNA was observed after beraprost administration, which might contribute to the down-regulation of the target pro-fibrotic genes. We noted that enhancement of Smad2 phosphorylation at 0.5 h and 2 h induced by TGF β stimulation was not attenuated by neither cipaprost nor beraprost, which might be attributed to the possibility that autocrine of TGF β increased in cardiac fibroblasts or latent TGF β was activate in medium [60]. But both two prostacyclin analogues significantly decreased Smad2 phosphorylation at 12 h and 24 h, which might be due to the weakening of autocrine of TGF β with time. Taken together, beraprost and cicaprost do not inhibit immediate Smad phosphorylation, but do inhibit long term Smad2 phosphorylation. The possible explanation for this is that beraprost and cicaprost do not inhibit the TGF β driven phosphorylation of Smad2, but rather act downstream of TGF β signaling pathway.

Previous studies show that the intracellular level of cAMP is enhanced as well as CREB phosphorylation after IP is activated [26], [27], [43], [61]. Interestingly, Ang II also phosphorylates CREB in the locus coeruleus-like cell line CATH.a neurons, HL-1 myocytes and cultured adult rat cardiac fibroblasts [62]–[64]. From our results, we have found that Ang II increased phosphorylation of CREB at Ser133 but not Ser142 and more phosphyration of CREB after beraprost treatment. Contrary to the pro-fibrosis of Ang II, anti-fibrosis effect was achieved by beraprost in our study. Further western blot for proteins from cytoplasm and nucleus respectively showed that Ang II increased phosphorylation of CREB in the cytoplasm while beraprost enhanced that in nucleus. Phosphorylation of CREB at Ser133 resulted in the recruitment of CBP, a transcriptional co-activator that was essential for CREB-mediated gene activation [30]. Expression of a non-phosphorylated CREB mutant suppressed the inhibitory effect of cicaprost on cardiac fibrosis [16]. Our co-immunoprecipitation assay verified that binding of CBP with CREB in nucleus was indeed increased after beraprost pre-treatment. Meanwhile the association between Smad and CBP was significantly increased after Ang II stimulation, while beraprost did decrease this association. Our findings suggested that phosphorylated CREB in nucleus evoked by beraprost might act as a functional antagonist of TGF β-smad signaling and antagonized TGF β-smad-mediated profibrotic effects by competitively binding with CBP. Beraprost did increase phosphorylation of CREB at the level of the nucleus, by diverting CBP to inhibit recruitment of CBP by the Smad complex. Altogether, inhibition of TGF β-Smad signal pathway might be responsible for the attenuation effects of beraprost on cardiac fibrosis.

**Figure 9:**
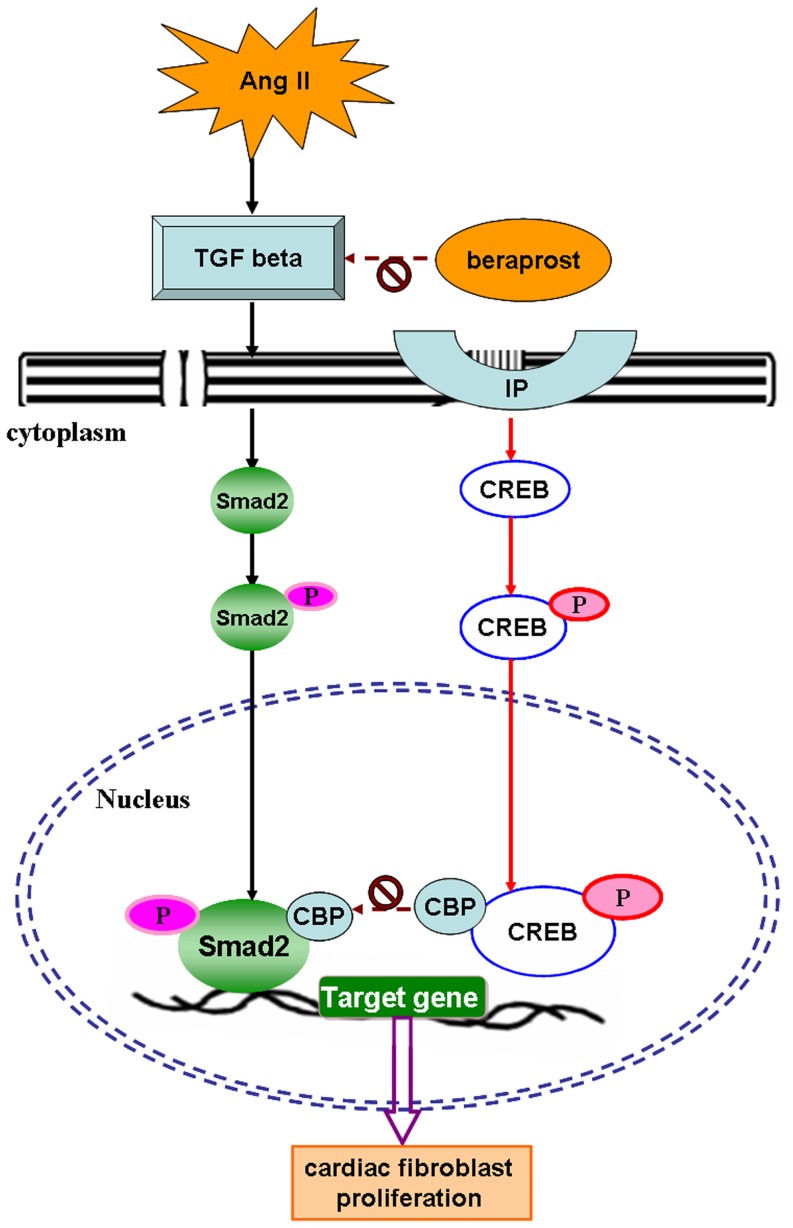
Illustration for mechanism of beraprost-mediated inhibition on cardiac fibroblast proliferation. In response to an Ang II stimulus, TGF β-Smad signal pathway is activated in cardiac fibroblasts. More phosphorylation of Smad2 translocates into nucleus to form a Smad2-CBP complex, which might facilitate fibrotic gene transcription and accelerate cardiac fibroblast proliferation. Beraprost binds to cell surface prostacyclin receptor (IP), phosphorylates of CREB at Ser133 and translocates into nucleus of cardiac fibroblasts, which sequestrates CBP and then prevents Smad-related transcription from performing an anti-fibrotic response. Beraprost may also inhibit TGF β-Smad signal pathway directly to attenuate cardiac fibroblast proliferation but the detailed mechanism needs further study.

In summary, our study suggests that prostacyclin analogue beraprost inhibited cardiac fibroblast proliferation via activation of IP but not PPAR, which might be related to a suppressive TGF β-Smad pathway. Beraprost increased phosphorylation and accumulation of CREB in the nucleus allowing it to bind with CBP and decreased Smad/CBP association to prevent Smad-related transcription to perform an anti-fibrotic response. An illustration for mechanism of beraprost-mediated inhibition on cardiac fibroblast proliferation was outlined (Fig. 9).
